# Nurse-Led Telephone Triage in Contemporary Healthcare: Bridging the Gap Between Patient Need and Resource Allocation

**DOI:** 10.3390/healthcare14040461

**Published:** 2026-02-12

**Authors:** Motti Haimi

**Affiliations:** 1 Health Systems Management Department, The Max Stern Yezreel Valley College, D.N Emek Yezreel, Mizra 1930600, Israel; mottih@yvc.ac.il; 2 Rappaport Faculty of Medicine, Technion-Israel Institute of Technology, Haifa 3109601, Israel

**Keywords:** nurse teletriage, telephone triage, telemedicine, patient safety, clinical decision support systems, healthcare access, quality assurance

## Abstract

**Background:** Nurse teletriage has emerged as a component of modern healthcare delivery, utilizing telecommunication technologies to assess patient conditions remotely and guide appropriate care decisions. As healthcare systems face increasing demand and the need for cost-effective care delivery, teletriage services have expanded, particularly following the COVID-19 pandemic. **Objective**: This narrative review examines the current state of nurse teletriage practice, its effectiveness, safety outcomes, and implementation considerations. A comparative analysis with physician-led teletriage models is provided, and the emerging role of artificial intelligence is explored. **Methods**: A narrative review of the literature was conducted through searches of multiple databases including PubMed/MEDLINE, CINAHL, Cochrane Library, Embase, Web of Science, and Google Scholar. This approach was selected due to the heterogeneous nature of the teletriage literature, which spans diverse study designs, populations, and outcomes that are not amenable to formal systematic synthesis. Peer-reviewed articles published between 1970 and 2024 examining safety outcomes, effectiveness, and implementation frameworks were reviewed. **Results**: The available evidence suggests that nurse-led teletriage systems, particularly when supported by computerized decision support systems, can improve patient access to care while maintaining safety standards. Studies indicate that telephone triage nursing does not increase mortality, hospitalization rates, or emergency department referrals when properly implemented. One well-documented physician-led model in Israel reported diagnosis accuracy rates of 98.5% and decision reasonableness rates of 92%, though generalizability across settings requires caution. Key success factors appear to include the use of evidence-based protocols, staff training, technology infrastructure, and quality assurance programs. While these findings are promising, the heterogeneous nature of the included studies and absence of formal quality assessment warrant cautious interpretation. **Conclusions**: Nurse teletriage appears to be an effective and safe approach to healthcare delivery that addresses challenges in modern healthcare systems. The choice between nurse-led and physician-led models should consider population complexity, case types, available resources, and economic factors. Artificial intelligence technologies offer potential opportunities to enhance teletriage, though careful validation is essential. Future research should focus on long-term outcomes, comparative effectiveness across healthcare systems, and rigorous evaluation of AI applications. **Highlights:** Telephone triage services, where nurses or physicians assess patients remotely and guide them to appropriate care, have become increasingly important in modern healthcare. This narrative review examines the evidence on nurse-led telephone triage, comparing it with physician-led models and exploring emerging technologies like artificial intelligence. The available evidence suggests that nurse-led systems, when supported by appropriate protocols and training, can safely improve patient access to care while reducing healthcare costs. Physician-led models may offer advantages for complex cases but at higher costs. While artificial intelligence shows promise for enhancing triage accuracy, current evidence specific to telephone triage remains limited. Healthcare organizations should carefully consider their population needs, available resources, and local context when implementing teletriage services.

## 1. Introduction

The landscape of healthcare delivery has undergone transformation in recent decades, driven by technological advances, evolving patient expectations, and mounting pressures on healthcare systems. Among the developments in this evolution is the emergence of teletriage services, representing a shift in how healthcare systems approach initial patient assessment and care coordination. Teletriage combines traditional clinical assessment skills with modern telecommunication technologies to address challenges facing contemporary healthcare systems [1].

### 1.1. Healthcare System Pressures and the Need for Innovation

Modern healthcare systems face challenges that demand innovative solutions, including increasing demand for services, growing primary care workloads, and emergency department overcrowding [2,3]. Different healthcare systems have adopted varying approaches to address these pressures. In the United States, fee-for-service models have driven interest in teletriage as a means of reducing costly emergency department visits. The United Kingdom’s National Health Service has implemented NHS 111, a nurse-led telephone triage system serving the entire population [4]. Nordic countries have developed centralized teletriage services integrated with their universal healthcare systems [5,6]. These diverse approaches reflect both system-specific constraints and opportunities for teletriage implementation.

### 1.2. Historical Evolution and Technological Integration

The concept of triage originated from military medicine and has evolved into an integral part of civilian healthcare [7]. While the integration of telecommunications technology with clinical assessment began in the 1970s, it was the development of structured protocols and computerized decision support systems in the 1990s that enabled teletriage to evolve into a formalized subspecialty with standardized practices [8,9]. Wheeler’s pioneering work in developing age-specific telephone triage guidelines established the foundations that continue to influence contemporary practice [10].

### 1.3. The COVID-19 Catalyst

The COVID-19 pandemic served as an unprecedented catalyst for rapid expansion of teletriage services [11]. Healthcare systems worldwide implemented remote care delivery models to maintain essential services while minimizing infection risk. However, this rapid deployment also revealed challenges, including concerns about rushed implementation without adequate quality assurance, digital divide issues affecting vulnerable populations, and inequalities in telehealth access among elderly and low-income populations [11]. These experiences highlight both the potential and the limitations of teletriage expansion under crisis conditions.

### 1.4. Diverse Implementation Models

Global implementation of teletriage has revealed variation in approaches, reflecting differences in healthcare system structures, regulatory environments, and cultural preferences [12]. Some systems have adopted nurse-led models supported by decision support systems and physician oversight [10,13], while others have implemented physician-led models relying on clinical expertise and autonomous medical decision-making [12,14,15]. Model selection should consider the following factors: patient population complexity, case acuity and types, available workforce and costs, and healthcare system structure and reimbursement mechanisms.

### 1.5. Economic and Policy Considerations

The economic implications of teletriage extend beyond cost reduction to encompass healthcare system efficiency and sustainability [2,3]. Teletriage services offer potential to redirect patients from emergency departments to more appropriate care settings. However, detailed cost-effectiveness data remain limited, and reimbursement policies vary significantly across healthcare systems. Successful implementation requires attention to regulatory frameworks, reimbursement policies, and quality assurance standards [11,12].

### 1.6. Review Objectives

This narrative review aims to (1) synthesize current evidence on the safety and effectiveness of nurse teletriage; (2) provide comparative analysis with physician-led models, with particular attention to contextual factors affecting generalizability; (3) examine the emerging role of artificial intelligence in teletriage, distinguishing current applications from future potential; and (4) identify key implementation considerations and directions for future research. By examining evidence from multiple healthcare systems, this review aims to provide insights for healthcare leaders, policymakers, and clinicians considering teletriage implementation.

### 1.7. Key Definitions

To ensure conceptual clarity and reduce heterogeneity in interpretation, the following operational definitions are used throughout this review:

Telephone triage refers to the process of assessing patient symptoms and determining care urgency through telephone communication, using structured protocols or clinical judgment to guide patients to appropriate care settings [12,16].

Nurse-led teletriage denotes telephone triage services delivered primarily by registered nurses, typically supported by computerized decision support systems (CDSS) and with physician backup available for complex cases [10,13].

Safety, in the context of this review, refers to the absence of adverse patient outcomes attributable to the triage process, including mortality, hospitalization, and emergency department visits that could have been prevented by earlier or different triage decisions [5,6].

Effectiveness, as used in this review, refers to the degree to which teletriage achieves its intended outcomes, including appropriate care allocation, patient satisfaction, healthcare resource utilization, and clinical outcomes [2,16].

## 2. Methods

### 2.1. Review Approach and Rationale

A narrative review approach was selected for this synthesis. This approach was chosen because the teletriage literature is characterized by substantial heterogeneity in study designs, populations, interventions, and outcomes, making formal systematic synthesis and meta-analysis inappropriate. The aim of this review is to provide a comprehensive overview and synthesis of current knowledge rather than a quantitative summary of effect sizes. This approach allows for integration of diverse evidence types while acknowledging the limitations inherent in narrative synthesis.

### 2.2. Literature Search

Literature was identified through searches of PubMed/MEDLINE, CINAHL, Cochrane Library, Embase, Web of Science, and Google Scholar. The final search was conducted in October 2024. Search terms included combinations of “nurse teletriage,” “telephone triage,” “telemedicine,” “patient safety,” “clinical decision-making,” and related terms. An example search strategy for PubMed was: (“telephone triage” OR “nurse teletriage” OR “telephone nursing”) AND (“patient safety” OR “clinical outcomes” OR “effectiveness”) AND (“decision support” OR “protocol” OR “guideline”). Google Scholar was included to identify seminal works and grey literature that may not be indexed in traditional databases, though this source’s limitations in search precision are acknowledged. The search focused on peer-reviewed articles published in English between 1970 and 2024.

### 2.3. Scope of Review

The review focused on studies examining nurse-led or physician-led teletriage systems, with attention to safety and effectiveness outcomes, implementation considerations, and comparative analyses. Inclusion criteria were (1) peer-reviewed articles published in English; (2) studies examining teletriage systems in developed healthcare settings with formal programs; (3) reporting of safety, effectiveness, or implementation outcomes. Exclusion criteria were (1) conference abstracts and editorials without original data; (2) studies focusing solely on in-person triage; (3) studies without clear methodology or outcomes reporting; (4) non-English publications. Systematic reviews, randomized controlled trials, observational studies, and qualitative research were considered.

### 2.4. Study Selection and Synthesis

Studies were identified through iterative searching, screening of titles and abstracts, and review of full texts for potentially relevant articles. Given the single-reviewer approach, potential selection bias is acknowledged as a limitation. Studies selected for detailed discussion were identified based on (1) landmark status in establishing foundational evidence; (2) recent comprehensive systematic reviews; (3) large sample sizes or rigorous methodology; and (4) provision of unique comparative data. This selection process involves inherent subjectivity, which is a recognized limitation of narrative reviews.

### 2.5. Quality Assessment

Consistent with narrative review methodology, formal quality assessment tools were not uniformly applied. Preference was given to systematic reviews and randomized controlled trials where available. Evidence was considered strongest when derived from systematic reviews and randomized controlled trials, moderate when from well-designed observational studies with adequate sample sizes, and limited when from single-system evaluations or qualitative studies. The absence of formal quality assessment is acknowledged as a limitation, and qualifying language is used throughout the Results and Discussion to reflect varying levels of evidence.

### 2.6. Synthesis Approach

A narrative synthesis approach was employed to summarize findings, identify key themes, and provide comparative analysis. Particular attention was paid to contextual factors affecting generalizability across different healthcare systems. Findings are presented thematically with appropriate caveats regarding the strength of evidence. Key studies were defined as those meeting one or more of the following criteria: (1) landmark studies that established foundational evidence in the field; (2) recent systematic reviews providing comprehensive synthesis of the literature; (3) studies with large sample sizes or particularly rigorous methodology; and (4) studies providing unique comparative data between nurse-led and physician-led models. This process of identifying key studies inherently involves some subjectivity, which is acknowledged as a limitation of narrative reviews.

## 3. Results

### 3.1. Overview of Evidence

The literature search identified studies spanning from the late 1970s to 2024, with the majority published after 2000, reflecting growing interest in teletriage research. Studies were conducted across multiple countries, with substantial representation from the United States, United Kingdom, Israel, Netherlands, and Australia. Study populations ranged from pediatric-specific services to general adult populations, with varying sample sizes from small qualitative studies to large national evaluations.

### 3.2. Safety and Effectiveness Outcomes

The literature generally indicates that well-implemented nurse teletriage systems can maintain safety standards [5,6,16]. Multiple systematic reviews have reported that telephone triage nursing does not appear to increase mortality rates, hospitalization rates, or emergency department referrals when implemented with appropriate protocols and decision support systems [4,6,16]. Studies comparing nurse-led teletriage with physician assessments suggest comparable safety outcomes when nurses use computerized decision support systems [17,18]. However, it should be noted that the quality of individual studies varies, and most evidence comes from observational designs. Findings supported by multiple systematic reviews (mortality, hospitalization, ED referrals) represent the strongest evidence in this review, while outcomes from single-system evaluations (such as the Israeli physician-led model) should be interpreted with greater caution regarding generalizability.

One physician-led pediatric teletriage service in Israel (Clalit Health Services) reported diagnosis accuracy rates of 98.5% and decision reasonableness rates of 92% [12,15]. While these results are noteworthy, the prominence of the Israeli model in this review reflects the availability of published research rather than an endorsement of this approach as universally superior. Limited published data exist on comparable physician-led models from other countries, which is a gap in the literature. Studies with mixed or negative findings have reported challenges including variable protocol adherence, concerns about over-triage in some settings, and difficulties with certain patient populations [19]. A summary of key safety and effectiveness findings is presented in Table 1.

### 3.3. Healthcare Utilization and Cost-Effectiveness

Evidence suggests positive impacts of teletriage on healthcare resource utilization [2,3]. Studies indicate reductions in emergency department utilization, with teletriage services redirecting patients to alternative care settings [3,11]. Cost savings of 20–40% have been reported primarily in UK general practice settings (ESTEEM trial) and Australian ambulance services; transferability of these estimates to other healthcare financing models requires caution [2,3]. ED redirection rates of 15–30% have been observed in Australian ambulance triage and UK NHS 111 services; actual rates vary substantially based on local healthcare infrastructure, patient populations, and triage protocols [3,4]. The ESTEEM trial, one of the largest randomized controlled trials in this field, indicated that telephone triage could manage same-day consultation requests in general practice while maintaining patient safety and satisfaction [2]. Cost-effectiveness analyses have generally favored teletriage implementation, though detailed economic data across different healthcare system contexts remain limited.

### 3.4. Technology Integration and Decision Support

The literature reveals evolution in decision support systems used in teletriage [17,20]. Early studies relied primarily on paper-based protocols, while contemporary research examines the role of computerized decision support systems (CDSS) in improving consistency and safety of triage decisions [18,21]. Studies examining artificial intelligence in triage settings show early promising results, though most AI research has been conducted in emergency department rather than telephone triage contexts [22]. The importance of maintaining human clinical judgment and ensuring appropriate validation of AI tools is consistently emphasized [12,22].

### 3.5. Workforce and Training Considerations

Research consistently highlights the importance of specialized training for teletriage personnel [23,24]. Studies examining nurse experiences reveal that telephone-based assessment requires specific skills including advanced communication, technological proficiency, and the ability to manage uncertainty in clinical decision-making [23,24]. Physicians in teletriage settings have reported initial difficulties with remote diagnosis but developed confidence through experience, structured protocols, and integration of non-medical factors in decision-making [14,15].

### 3.6. Gaps in the Literature

Notable gaps include limited long-term outcome studies, insufficient research on AI integration in telephone triage specifically (as opposed to ED triage), inadequate comparative data between different teletriage models across healthcare systems, and limited research on teletriage applications in chronic disease management. Most studies focus on acute care applications in developed healthcare systems.

## 4. Discussion

### 4.1. Definition and Scope of Nurse Teletriage

Nurse teletriage involves remote assessment of patients’ health conditions through telecommunication technologies to determine care urgency and appropriate settings [16,23]. Recent research defines teletriage as “the complex process of remotely assessing acute, worrisome symptoms to estimate their urgency and to render clinical advice and triage for further evaluation and diagnosis, as appropriate” [12]. This process enables qualified nurses to evaluate symptoms, provide health education, and guide patients toward appropriate care levels. The scope extends across multiple healthcare settings, including primary care, emergency departments, and occupational health services [11,19].

### 4.2. Current Evidence and Effectiveness

Available research suggests that nurse teletriage systems can maintain safety standards while improving healthcare accessibility [6,16]. Studies indicate that nurses using computerized decision support systems may achieve quality and safety rates comparable to general practitioners [17,18]. The 24/7 availability of teletriage services appears particularly valuable for individuals requiring after-hours medical guidance [4,9]. The ESTEEM trial provided evidence that telephone triage could effectively manage same-day consultation requests [2]. However, findings should be interpreted cautiously given the observational nature of much of the evidence and heterogeneity across settings.

### 4.3. Physician Teletriage: A Comparative Perspective

While nurse-led teletriage is the predominant model in many systems, physician-led teletriage offers an alternative approach. The fundamental difference lies in clinical training and autonomous decision-making capabilities [12]. Israel provides an example through services such as the Clalit Pediatricians Online Service [12,14,15]. The UK has implemented GP-led telephone triage in some primary care settings, and Nordic countries have developed nurse–physician collaborative models. Each approach reflects local healthcare system structures and workforce availability.

Physician-led teletriage may offer advantages for complex cases requiring advanced clinical judgment, though at higher costs [12,15]. However, physicians also face challenges including difficulty diagnosing without physical examination and managing uncertainty in remote settings [14]. For general populations with common acute conditions, well-structured nurse-led systems with appropriate decision support may provide adequate safety at lower costs. The choice between models should depend on population complexity, case types, available resources, and healthcare system structure. A comparison of nurse-led and physician-led models is presented in Table 2.

### 4.4. The Role of Artificial Intelligence in Teletriage

Artificial intelligence represents a promising technology with the potential to enhanc teletriage, though it is important to distinguish between current clinical applications and future possibilities. Most AI triage research has been conducted in emergency department settings rather than telephone triage specifically [22]. The evidence base for AI applications in telephone-based nurse triage remains limited, and findings from ED settings may not directly translate to telephone triage contexts.

Potential AI applications include natural language processing for symptom analysis, machine learning for risk stratification, and predictive modeling for clinical outcomes. However, significant challenges must be addressed, including: validation gaps (most AI tools have not been rigorously tested in real-world telephone triage settings); algorithmic bias (AI systems may perform differently across patient populations); legal and liability considerations (unclear responsibility when AI contributes to clinical decisions); data privacy concerns; and risks of over-reliance on AI recommendations at the expense of clinical judgment.

The implementation of AI in teletriage should be approached cautiously. AI systems require rigorous testing and validation specific to telephone triage settings before deployment. Clinicians must understand both the capabilities and limitations of AI tools [23,24]. Healthcare organizations should ensure that patient safety remains paramount and that AI enhances rather than replaces clinical judgment [4,11]. Current and potential AI applications in teletriage are summarized in Table 3.

### 4.5. Implementation Considerations

Successful implementation of nurse teletriage systems requires attention to staffing, training, technology, and quality assurance [23,24]. Nurses conducting teletriage need strong assessment skills, communication abilities, and familiarity with telecommunication technologies. Specialized training in telephone-based assessment techniques and computerized decision support systems appears essential [11,20]. Effective teletriage also requires robust technology infrastructure including reliable telecommunication systems, secure data transmission, and integrated decision support tools [1,17]. Comprehensive quality assurance programs including monitoring of triage decisions, outcomes tracking, and continuous improvement processes are important for maintaining effectiveness and safety [11,19]. Critical implementation success factors are summarized in Table 4.

### 4.6. Challenges and Limitations of Teletriage Practice

Nurse teletriage faces inherent limitations related to the inability to conduct physical examinations or observe visual cues [23,24]. These limitations require reliance on verbal communication and structured protocols. Research has documented challenges in making decisions under uncertainty [14]. Healthcare organizations must consider liability and legal implications, establishing clear policies, documentation requirements, and escalation protocols [12,13]. Patient acceptance and effective communication are also important, with some patients preferring in-person interactions or facing difficulty communicating symptoms by telephone [23,25].

### 4.7. Future Directions

The future of nurse teletriage may involve greater integration with digital health technologies, including remote monitoring devices and mobile health applications [1,22]. Teletriage may expand into new service models, including chronic disease management and specialized care coordination may occur [3,9]. Growing demand necessitates focused workforce development including specialized education and certification opportunities [23,24]. Research priorities should include long-term outcome studies, rigorous AI evaluation specific to telephone triage, and comparative effectiveness research across healthcare systems.

### 4.8. Limitations of This Review

This narrative review has several important limitations. First, the absence of formal systematic search methodology and quality assessment means that study selection involved subjectivity and potential bias. Second, the single-reviewer approach may have introduced selection bias. Third, the evidence base is geographically limited, with most studies from developed Western healthcare systems, limiting generalizability to other contexts. Fourth, the Israeli physician-led model is prominently featured due to available published research, but this should not be interpreted as evidence of superiority; comparable data from other physician-led systems are limited. Fifth, the heterogeneous nature of included studies (varying designs, populations, interventions, and outcomes) limits the strength of conclusions that can be drawn. Finally, the absence of formal quality appraisal means that findings from methodologically weaker studies may have been given similar weight to stronger evidence. These limitations should be considered when interpreting the findings of this review.

## 5. Conclusions

Based on the available evidence, several conclusions can be drawn with varying degrees of confidence.

- Well-supported by consistent evidence: Nurse teletriage, when implemented with appropriate protocols, training, and decision support systems, appears to be a safe approach that does not increase adverse outcomes compared to traditional care pathways.
- Supported by moderate evidence: Teletriage can improve healthcare access and may reduce costs, though detailed economic data across different healthcare contexts remain limited. Comparative evidence suggests that both nurse-led and physician-led models can achieve good outcomes, with choice depending on population needs, case complexity, available resources, and system structure.
- Emerging evidence requiring further confirmation: Artificial intelligence technologies show potential to enhance teletriage, but current evidence specific to telephone triage is limited and primarily extrapolated from emergency department research.
- Areas where evidence remains limited: Long-term patient outcomes, comparative effectiveness across different healthcare systems, cost-effectiveness in diverse contexts, and optimal integration of AI tools all require further research.

Healthcare organizations considering teletriage implementation should carefully plan their approach based on local context, invest in appropriate training and technology, and establish quality assurance measures. The experiences from different healthcare systems offer guidance, but findings may not be directly transferable across settings. Continued research and evaluation will be essential to optimize teletriage services and their integration with evolving healthcare delivery models.

## Figures and Tables

**Table 1:** Summary of Key Safety and Effectiveness Findings.

Outcome Measure	Finding	Evidence Strength	Healthcare Context	Source
Mortality rates	No increase with proper implementation	Multiple systematic reviews	UK, Netherlands, multiple countries	[5,6,16]
Hospitalization rates	No increase observed	Systematic reviews	UK NHS, European systems	[4,6,16]
ED referral rates	Appropriate when properly implemented	RCT	UK out-of-hours primary care	Lattimer [16]
Diagnosis accuracy	98.5% (physician-led)	Single-system evaluation	Israeli HMO pediatric service	Haimi [12,15]
Cost savings	20–40% reduction	Limited economic analyses	UK general practice, Australian ambulance	[2,3]
ED redirection rate	15–30% redirected	Multiple observational	Australian ambulance, UK NHS 111	Eastwood [3]

**Table 2:** Comparison of Nurse-Led vs. Physician-Led Teletriage Models.

Characteristic	Nurse-Led Model	Physician-Led Model
Primary decision support	Computerized protocols (CDSS)	Clinical expertise + protocols
Diagnosis accuracy	High with CDSS support	98.5% (Israeli model)
Decision reasonableness	Comparable to GPs with CDSS	92% (Israeli model)
Cost-effectiveness	Higher (lower personnel costs)	Lower (higher physician salaries)
Training requirements	Specialized teletriage training	Medical education + adaptation
Best suited for	General populations, common conditions	Complex cases, pediatric specialty
Supervision needed	Physician oversight recommended	Autonomous decision-making
Scalability	High	Moderate (physician availability)

**Table 3:** AI Applications in Teletriage.

Application	Current Status	Potential Benefits	Key Challenges
Natural language processing	Emerging (mostly ED research)	Automated symptom analysis	Validation in phone triage
ML risk stratification	Pilot implementations	Improved urgency classification	Algorithmic bias
Predictive outcome modeling	Research phase	Better clinical decision support	Legal/liability issues
Wearables integration	Early adoption	Objective physiological data	Data privacy concerns
Pattern recognition	Promising early results	Identification of high-risk cases	Over-reliance risk

**Table 4:** Critical Implementation Success Factors.

Factor	Description	Key Considerations
Staffing	Qualified personnel with specialized skills	Strong assessment, communication abilities
Training	Comprehensive teletriage education	Phone techniques, CDSS use, uncertainty management
Technology	Robust infrastructure	Reliable telecom, secure data, integrated CDSS
Protocols	Evidence-based standardized guidelines	Schmitt-Thompson, Wheeler guidelines
Quality assurance	Continuous monitoring programs	Outcomes tracking, performance metrics
Oversight	Appropriate supervision structure	Physician backup, escalation protocols

## Data Availability

No new data were created or analyzed in this study.
